# The burden of diabetes and hyperglycemia in Brazil: a global burden of disease study 2017

**DOI:** 10.1186/s12963-020-00209-0

**Published:** 2020-09-30

**Authors:** Bruce Bartholow Duncan, Ewerton Cousin, Mohsen Naghavi, Ashkan Afshin, Elisabeth Barboza França, Valéria Maria de Azeredo Passos, Deborah Malta, Bruno R. Nascimento, Maria Inês Schmidt

**Affiliations:** 1 Programa de Pós-graduação em Epidemiologia e Hospital de Clínicas de Porto Alegre, Universidade Federal do Rio Grande do Sul, R. Ramiro Barcelos, 2600 Sala 414, Porto Alegre, RS 90035-003 Brazil; 2 Institute for Health Metrics and Evaluation, University of Washington, Seattle, USA; 3 Programa de Pós-graduação em Saúde Pública, Universidade Federal de Minas Gerais, Belo Horizonte, Brazil; 4 Faculdade Ciências Médicas de Minas Gerais, Belo Horizonte, Brazil; 5 Departamento de Enfermagem Materno-Infantil e Saúde Pública, Escola de Enfermagem, Universidade Federal de Minas Gerais, Belo Horizonte, Brazil; 6 Hospital das Clínicas da Universidade Federal de Minas Gerais, Belo Horizonte, Brazil

**Keywords:** Diabetes mellitus, hyperglycemia, epidemiology, Global Burden of Disease, Brazil

## Abstract

**Background:**

The Global Burden of Diseases (GBD) 2017 database permits an up-to-date evaluation of the frequency and burden of diabetes at the state level in Brazil and by type of diabetes. The objective of this report is to describe, using these updated GBD data, the current and projected future burden of diabetes and hyperglycemia in Brazil, as well as its variation over time and space.

**Methods:**

We derived all estimates using the GBD 2016 and 2017 databases to characterize disease burden related to diabetes and hyperglycemia in Brazil, from 1990 to 2040, using standard GBD methodologies.

**Results:**

The overall estimated prevalence of diabetes in Brazil in 2017 was 4.4% (95%UI 4.0–4.9%), with 4.0% of those with diabetes being identified as having type 1 disease. While the crude prevalence of type 1 disease has remained relatively stable from 1990, type 2 prevalence has increased 30% for males and 26% for females. In 2017, approximately 3.3% of all disability-adjusted life years lost were due to diabetes and 5.9% to hyperglycemia. Diabetes prevalence and mortality were highest in the Northeast region and growing fastest in the North, Northeast, and Center-West regions. Over this period, despite a slight decrease in age-standardized incidence of type 2 diabetes, crude overall burden due to hyperglycemia has increased 19%, with population aging being a main cause for this rise. Cardiovascular diseases, responsible for 38.3% of this burden in 1990, caused only 25.9% of it in 2017, with premature mortality attributed directly to diabetes causing 31.6% of the 2017 burden. Future projections suggest that the diabetes mortality burden will increase 144% by 2040, more than twice the expected increase in crude disease burden overall (54%). By 2040, diabetes is projected to be Brazil’s third leading cause of death and hyperglycemia its third leading risk factor, in terms of deaths.

**Conclusions:**

The disease burden in Brazil attributable to diabetes and hyperglycemia, already large, is predicted by GBD estimates to more than double to 2040. Strong actions by the Ministry of Health are necessary to counterbalance the major deleterious effects of population aging.

## Background

The prevalence and burden of diabetes mellitus are rising worldwide [1, 2]. The burden in Brazil, which has the fourth largest absolute number of cases of diabetes in the world [2], is no exception. Many public health options exist to confront this problem [3]. With the renewal of executive administrations at both Brazilian federal and state levels in January 2019, new teams have assumed the public health responsibility of protecting the population’s health. A review of the burden due to diabetes and hyperglycemia and projected future trends of these burdens at this point is thus timely.

The Global Burden of Disease Project (GBD), the world’s leader in the epidemiology of disease burden, has recently released its 2017 estimates. Recent GBD methodologic advances now permit characterization of the frequency and burden of diabetes at the state level in Brazil and by type of diabetes. The GBD also offers, for the first time, projections of future trends in burden.

The objective of this report is to describe, using these updated GBD data, the current and projected future burden of diabetes and hyperglycemia in Brazil, as well as its variation over time and space.

## Methods

Our analyses produce estimates using the GBD 2017 database to characterize disease burden related to diabetes and hyperglycemia in Brazil from 1990 to 2017. The GBD project generates a series of indicators of disease burden around the world by summarizing data on the frequency of risk factors, diseases, and their complications together with estimates of the interrelations of these elements which it derives from systematic reviews of the literature. Access to these data is provided by the infographic GBD Compare 2017 [4] and the GBD Results Tool [5], which include multiple assessments of disease burden and its component parts covering the period from 1990 to 2017. We additionally used the new infographic GBD Foresight [6], which offers projections of some components of disease burden up to 2040 based on the GBD 2016 database. Detailed descriptions of methodologies and approaches of the framework developed to produce these data have been published elsewhere [2, 7–10].

The current GBD framework maintains the approach of recognizing diabetes, now broken down into type 1 and type 2, both as a disease with its proper complications, as a distinct cause of chronic kidney disease, and as one of the multiple contributing causes of a series of other diseases [11]. This latter expression of its pathology is accounted for through the risk factor category of high fasting plasma glucose, which also encompasses the effects of lower levels of hyperglycemia [10]. Though denominated high fasting plasma glucose, this metric integrates data from diverse measures of glycemia. Supplementary Figure 1 summarizes this GBD combined burden approach. Burden ascribed directly to diabetes is defined through ICD codes E10-13, except the “.2” codes, which relate to renal disease—and includes that due to living with diabetes (“uncomplicated diabetes”), to premature deaths from both acute and chronic complications which are coded to diabetes, and to disability caused by its traditional “microvascular” complications (vision loss, including severe low vision and blindness, and neuropathy, including diabetic foot and amputation) [8, 12]. This direct burden is shown in the part of the figure enclosed by the dotted red line. Chronic kidney disease due to diabetes is treated as a separate item. High fasting plasma glucose is used to account for the rest of the burden. This additional part derives from the recognized complications of both diabetes and lesser hyperglycemia, which, while not coded in underlying data sources as due to diabetes, have been demonstrated in the literature to result in part from diabetes or lesser hyperglycemia. It includes cardiovascular diseases (ischemic heart disease, ischemic stroke, hemorrhagic stroke), chronic kidney disease, dementia, respiratory infections and tuberculosis, and sense organ diseases such as cataract and age-related hearing loss [9]. In GBD calculations of these burdens, the theoretical minimum risk exposure level of glucose is taken to be 4.8–5.4 mmol/L (86.4–97.2 mg/dl).

The GBD uses four main indicators to calculate disease burden—mortality, years of life lost due to premature mortality (YLLs), years of life lived with disability (YLDs), and the sum of the latter two–disability-adjusted life years (DALYs). Briefly, YLLs were calculated multiplying the number of deaths from diabetes or due to high fasting plasma glucose in each age group by the reference life expectancy at the average age of death for those who die in that age group [8]. YLDs were obtained initially for each complication of diabetes or of the other diseases resulting from hyperglycemia by multiplying the complication’s prevalence times its disability weight in each age, sex, and year specific strata, with total YLDs, then being aggregated across strata and complications [8]. These disability weights were derived from population-based surveys of the general public [12]. All results are standardized to the world population. Results are presented overall for Brazil and in specific instances separately for each of its federative units (states and the Federal District, hereafter called “states”).

Prediction of deaths due to diabetes from 2017 to 2040 was produced with a forecasting model applied to GBD 2016 data. Three results are presented—the best (“reference”) forecast and two alternative scenarios of better and worse health. These alternatives were generated by applying the 85th and 15th percentile annualized rates of change in deaths due to diabetes as observed across all locations in past years to diabetes deaths in Brazil, as described in greater detail elsewhere [13].

The study GBD Brazil was approved by the Research Ethics Committee of the Universidade Federal de Minas Gerais, Project CAAE—62803316.7.0000.5149.

## Results

### Incidence and prevalence

**Fig. 1:**
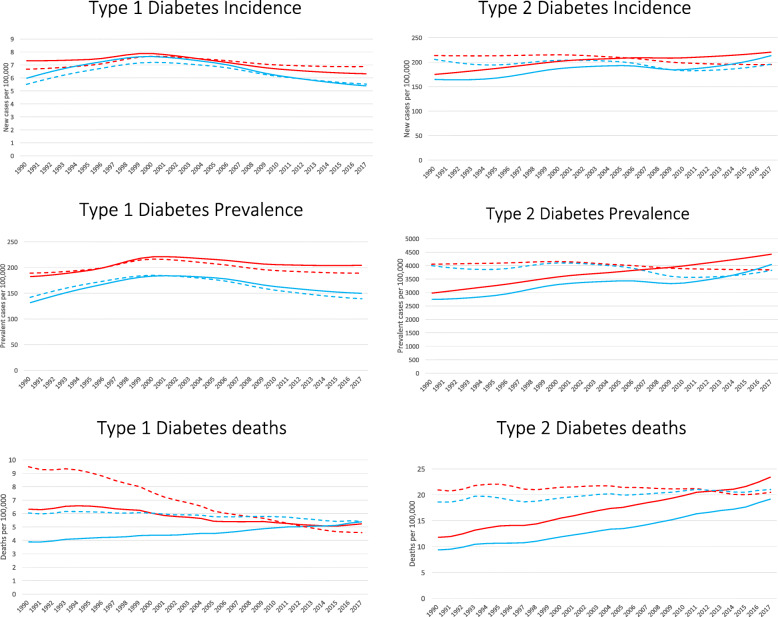
Trends in estimated incidence, prevalence, and mortality of type 1 (left) and type 2 (right) diabetes, Brazil, 1990–2017. Diabetes incidence (new cases/100,000 population; top), prevalence (cases/100,000 population; middle), and mortality (diabetes deaths/100,000 population; bottom). Solid lines: crude prevalence; dashed lines: age-standardized prevalence. Red lines: females, blue lines: males

The prevalence of diabetes, considering all ages in 2017, was 4.4% (95%UI 4.0–4.9%), being 6.2% for those aged 20 or above. Of those with diabetes, 4.0% were identified at type 1 and 96.0% as type 2. Figure 1 displays trends, from 1990 to 2017, of crude (solid lines) and age-standardized (dashed lines) incidence and prevalence of both types of diabetes. The estimate of crude incidence of type 1 disease (left top panel), though relatively stable considering the whole period, has been falling for both males (blue lines) and females (red lines) since approximately the year 2000. The crude prevalence estimate of type 1 disease (left middle panel) has also decreased since this date, especially in men. On the other hand, for type 2 diabetes, the estimates of crude incidence (right top panel; males up 48%, females up 49%) and prevalence (right middle panel; males up 30%, females up 26%) have risen considerably over the same period.

The equivalent age-standardized trends are also shown in Fig. 1. While the age-standardized estimates of incidence and prevalence of type 1 disease are not much different from the crude ones, that of age standardized incidence of type 2 diabetes has been slowly and irregularly declining since 1990, as has that of its age standardized prevalence.

**Fig. 2:**
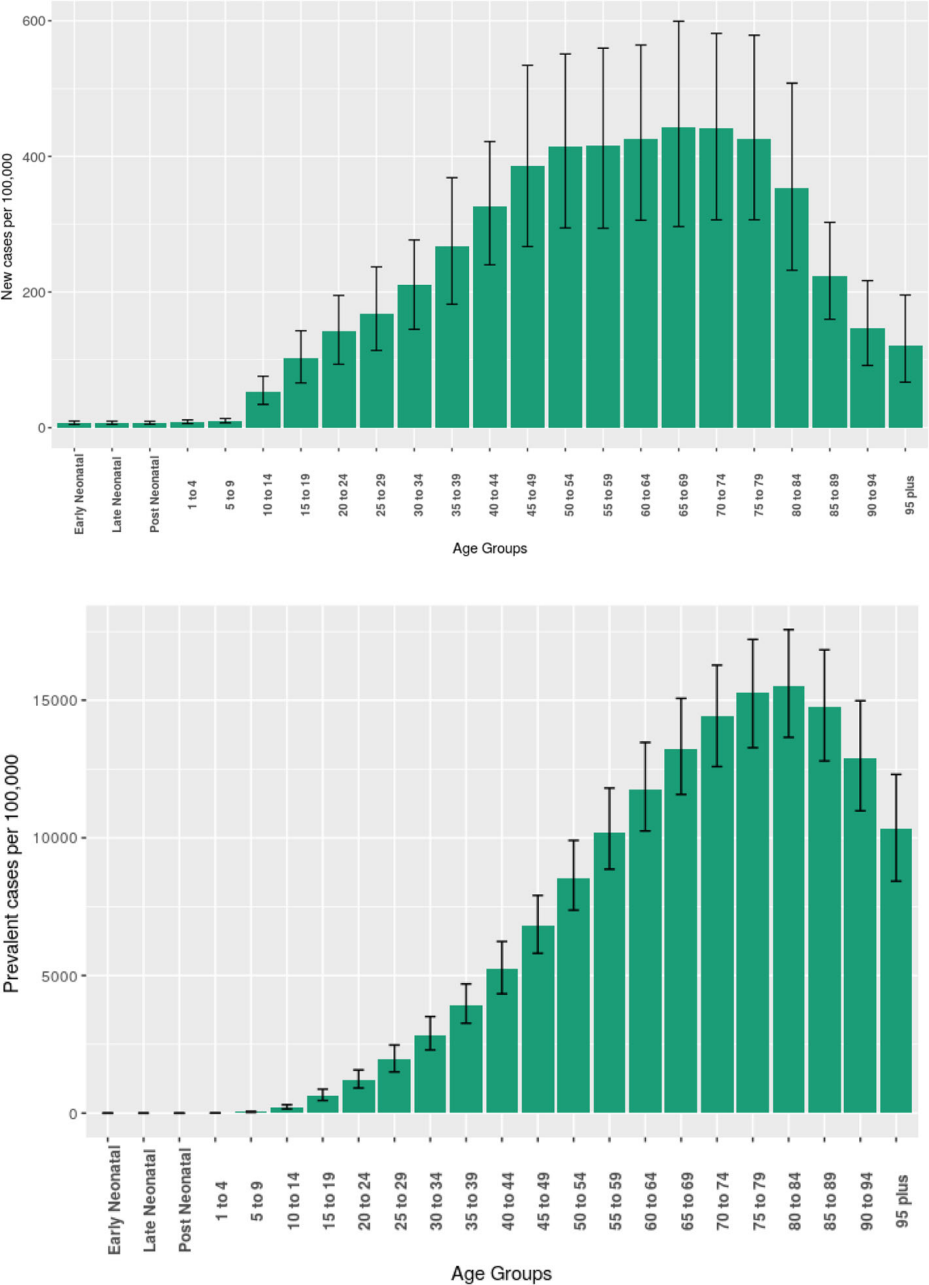
Estimated age-specific incidence (top) and prevalence (bottom) rates of diabetes, with their 95% uncertainty intervals. Brazil, 2017

The steep increase with age in estimates of diabetes incidence (left panel) and prevalence (right panel) seen in Fig. 2 explains why population aging has had such an important role in the evolution of the metrics seen in Fig. 1 from 1990 to present.

**Fig. 3:**
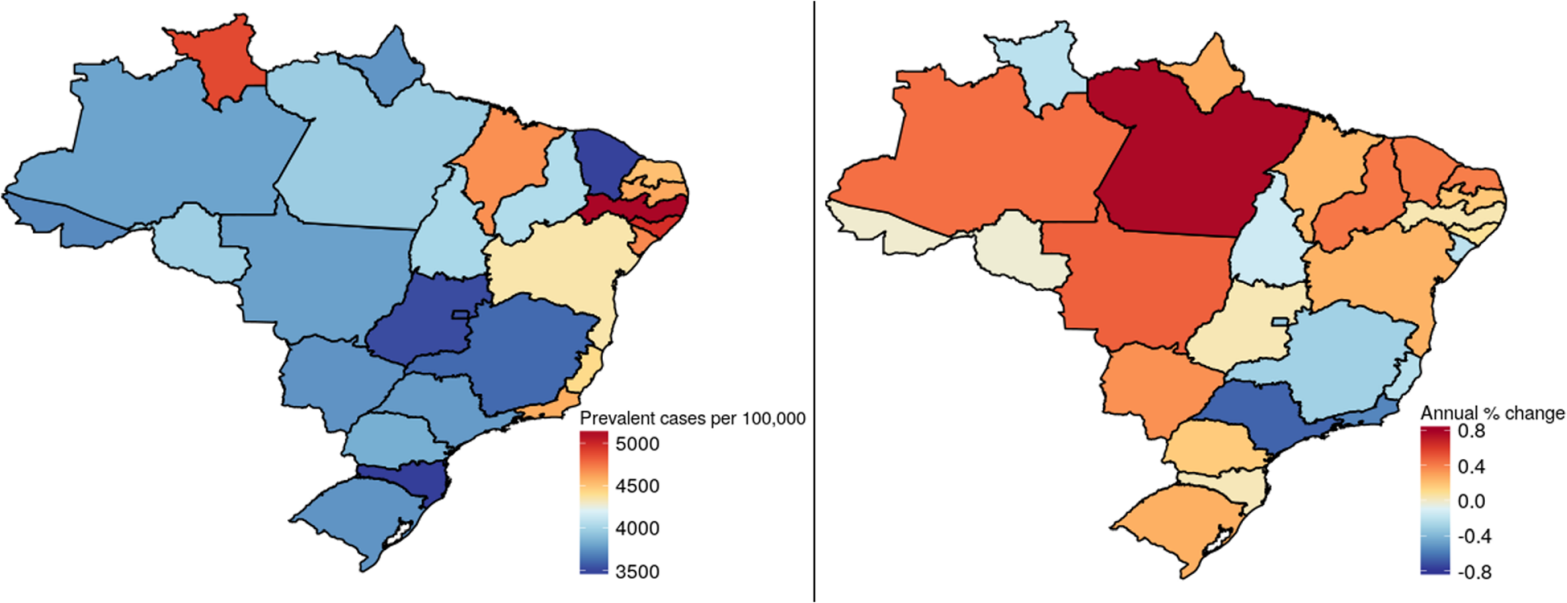
Prevalence and annual change in prevalence of diabetes mellitus in Brazilian states. Left panel: age-standardized prevalence (/100000 population), 2017. Right panel: annual change (%) in this prevalence, 1990–2017. The closer to the red end of the color scale, the greater the prevalence or increase in prevalence; the closer to the dark blue end, the lesser the prevalence or increase in prevalence

The estimated prevalence of diabetes demonstrated considerable geographic variability within Brazil. The map in the left panel of Fig. 3 shows that in 2017, states in the Northeast present a generally higher prevalence of diabetes (red to beige colors) than those of other regions. The right panel of this Figure shows that, over the past 27 years, states in the North, Northeast, and Center-West have suffered greater percentage increases in prevalence (red to beige colors), while for other states—notably those in the Southeast—prevalence has decreased (blue colors).

### Burden of disease

In 2017, premature mortality and disabilities from all diseases combined caused a loss of 28,556 (95%UI 25,689–31,888) DALYs/100,000 Brazilian population. Of these, 3.3% (938.5, 95%UI 802.9–1093.6/100000 population) were lost to diabetes in 2017, with type 1 disease being responsible for 16.8% of this total. This percentage increases to 5.9% (1683.4, 95%UI 1440.7–1945.6/100000 population) of total expected DALYs lost, when considering the broader category of diseases/ complications resulting from diabetes and lesser hyperglycemia when they are characterized together as high fasting plasma glucose.

The bottom panels of Fig. 1 show trends for one important aspect of this burden—mortality. For type 1 disease, age-standardized mortality over the period was estimated to decrease over 50% for females. For males, this decrease was less, only about 10%, thus approximating gender-specific rates that were quite different at the beginning of the period. Age-standardized rates for type 2 disease, in contrast, were basically stable over the period, with a slight decrease for women and a slight increase for men. When translated into crude rates, however, mortality for type 2 disease increased dramatically, basically doubling over the period.

**Fig. 4:**
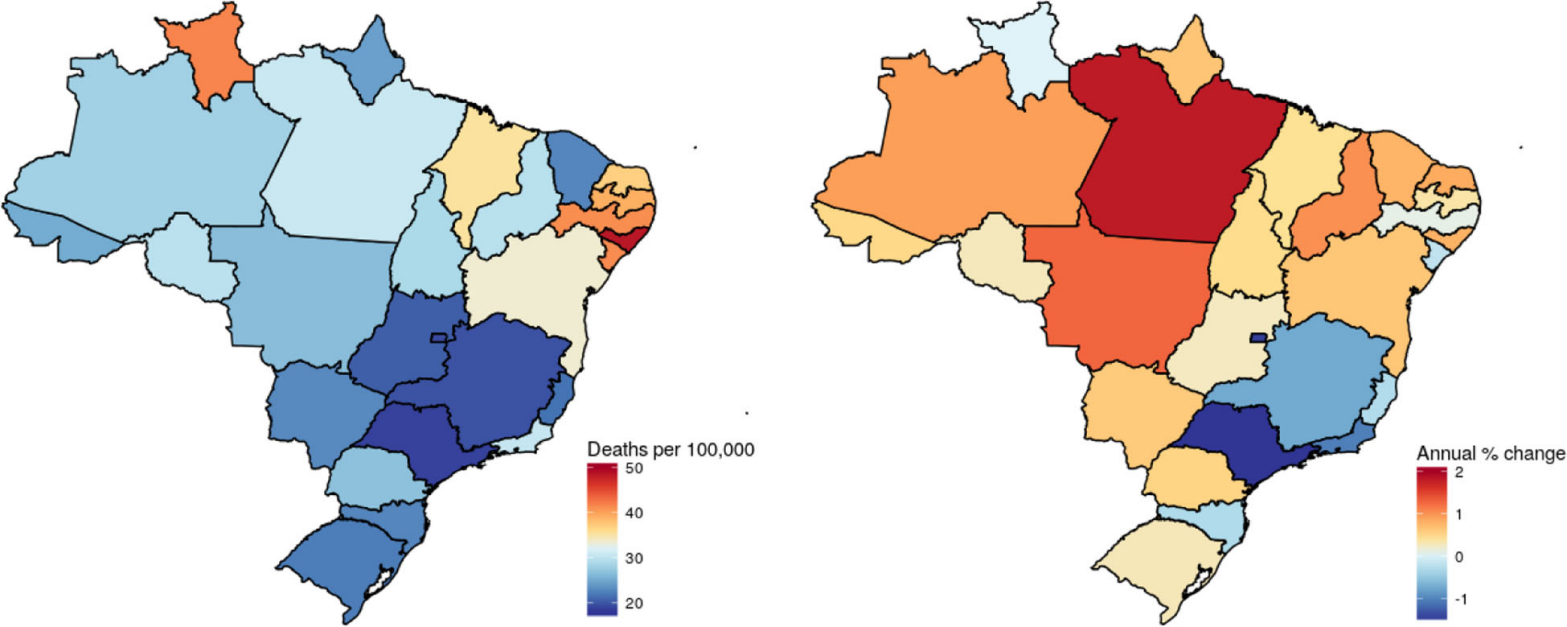
Mortality and annual changes in mortality due to diabetes mellitus in Brazilian states. Left panel: age-standardized mortality (/100000 population), 2017. Right panel: annual change (%) in this rate, 1990–2017. The closer to the red end of the color scale, the greater the mortality or increase in mortality; the closer to the dark blue end, the lesser the mortality or increase in mortality

Figure 4 shows the geographic distribution of age-standardized mortality due to diabetes, independent of type. Not surprisingly, the age-standardized rate of mortality and its trend both show geographic patterns quite similar to those of prevalence and change in prevalence. The greatest diabetes mortality rates (similarly, red to beige colors) in 2017 occurred principally in states of the Northeast. Greatest increases in mortality from 1990 to 2017 were seen in the North, Northeast, and Center-West, with greatest decreases (again, blue colors) seen in the Southeast.

**Fig. 5:**
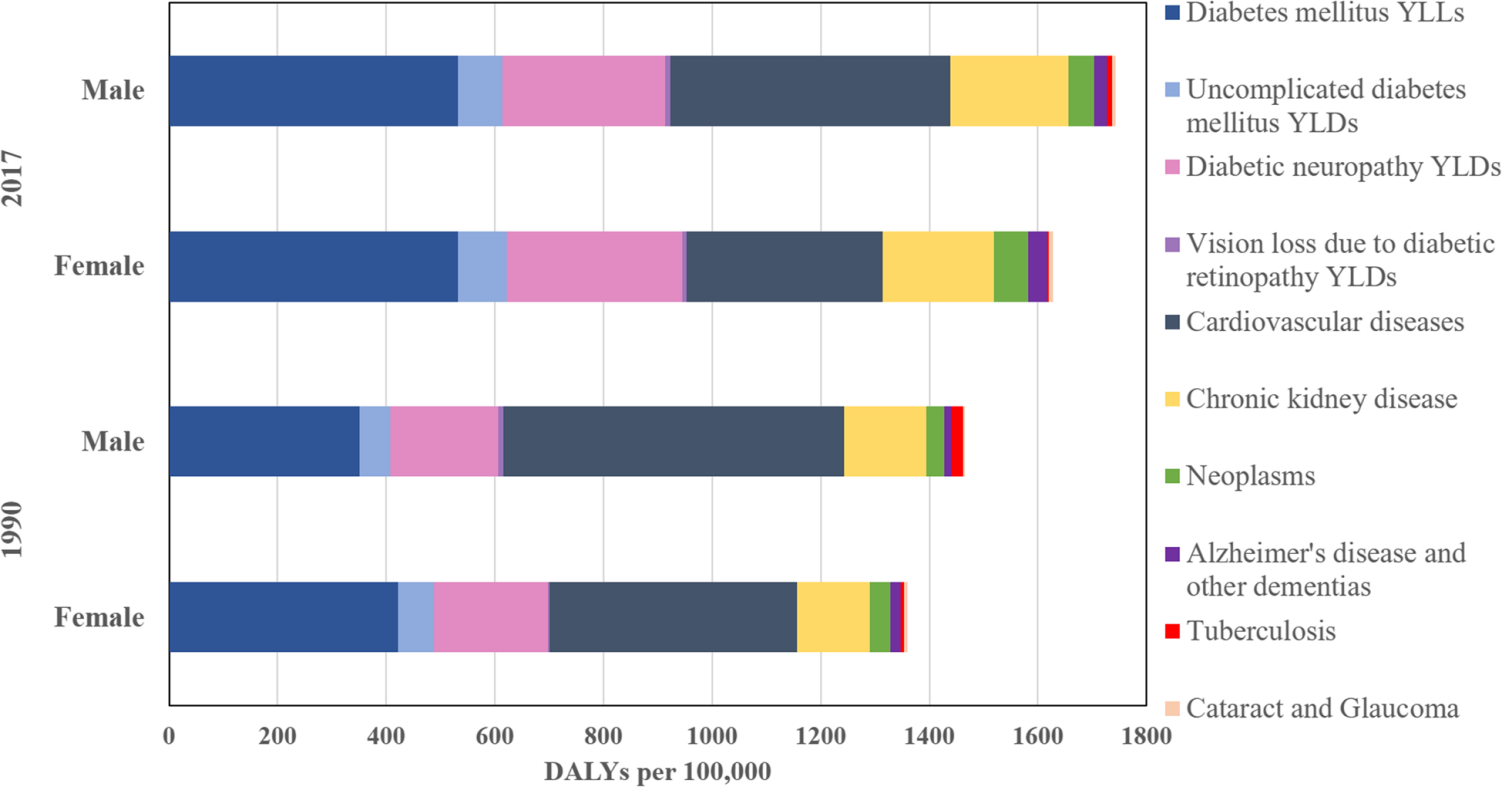
Crude burden (DALYs/100000 population) due to hyperglycemia in Brazil, by cause or type of complication, 1990 and 2017. When indicated, the category of burden reports only premature deaths (YLLs) or only disability (YLDs)

Figure 5 shows the causes (other diseases and complications) and size of the crude burden of diabetes/hyperglycemia in 1990 and 2017. Over this period, the overall diabetes burden has increased 19%, considering both males and females. Additionally, the causes of the burden have shifted considerably. Cardiovascular diseases represent a much smaller fraction of the total (decreasing from 38.3 to 25.9%), while chronic kidney disease (up from 10.1 to 12.6%), neoplasms (from 2.5 to 3.3%), premature deaths due to diabetes (from 27.4 to 31.6%), and disability resulting from diabetic neuropathy (14.5 to 18.5%) and living with diabetes (from 4.4 to 5.1%) have increased considerably.

When age-standardized, the total rate of DALYs lost due to high fasting plasma glucose decreased 29% (data not shown). These findings are similar to what was seen for prevalence, demonstrating that the increased mortality due to hyperglycemia results from population aging rather than to an increase in the underlying rates of burden.

**Fig. 6:**
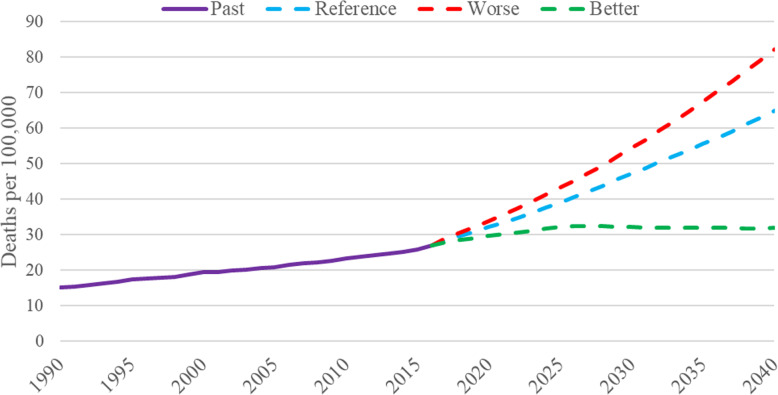
Past trends (1990–2016) and future projections (2017–2040) of overall crude mortality due to diabetes mellitus. Projections are given for reference and better and worse case scenarios

Figure 6, using the new infographics tool GBD Foresight, shows not only past trends in mortality due to diabetes but also projections in trends up to 2040. As can be seen from the graph, the crude mortality rates for both sexes, given projected aging of the population, are projected to increase dramatically, 127%, in the reference scenario. In the worst case scenario, the increase is considerably greater. In the better case scenario, the death rate estimates will stabilize around 2025. In comparison, the expected increase in crude mortality from all causes of disease burden is only 54% over this period of time. Not surprisingly, based on these GBD estimates, diabetes mellitus is projected over the next 23 years to rise from the sixth to the third leading cause of deaths and high fasting plasma glucose from the fourth to the third leading risk factor in terms of mortality.

## Discussion

These updated GBD analyses provide a comprehensive picture of the diabetes burden in Brazil. While some improvement has occurred in its age-standardized frequency and burden, population aging has resulted in increases in its crude incidence, prevalence, and burden from 1990 to 2017. Over the period studied, the growth in diabetes frequency and burden has been greatest in the Northeast, North, and Center-West regions, with concomitant decreases occurring in the Southeast region. The period studied has also witnessed an ongoing change in the causes of burden of hyperglycemia, with a greater spread of burden across disease/complication groupings and less concentration in cardiovascular diseases. Projections to the year 2040 suggest that the trend for an ever-greater burden due to diabetes and hyperglycemia will exacerbate, again driven principally by further aging of the population.

GBD estimates of prevalence are based primarily on Brazilian survey data, specifically those using blood testing. However, these data are sparse and sub-national and must thus be interpreted with caution. Estimates of the prevalence of diabetes in Brazil, not incorporated into the GBD due to being based on self-report or on medication use without accompanying lab testing, include self-report of diabetes from PNAD studies (1998, 2003, 2008) [14] and the National Health Survey (PNS) of 2013. The latter showed a national prevalence of 6.2% (5.9–6.6%) for those 18 or older, [15] identical to that found for those over 20 by the GBD. However, when defined as either a high glycated hemoglobin or medication use in a representative sub-sample undergoing blood testing of the PNS, diabetes prevalence increased to 8.4% (7.6–9.1%) [16]. Vigitel provides yearly estimates for Brazilian capitals from 2006 onward. Based on Vigitel data adjusted for age, BMI, and educational attainment, the prevalence of diabetes among adults has risen from 5.7 to 8% from 2006 to 2014, different from the slight decline in age-standardized prevalence shown here [17]. Additionally, GBD may also underestimate the true prevalence of diabetes since most surveys reporting laboratory results are based only on fasting glycemia and thus not accounting for cases detected only by an OGTT [18]. Moreover, given the doubling of the GBD’s summary exposure value for age-standardized high body mass index in Brazil over the period studied, a decline in age-standardized diabetes incidence and prevalence seems unlikely. Our GBD estimates thus should perhaps be seen as conservative ones. Further, though these estimated rates are declining overall, as shown in Fig. 3, they are rising in much of the country, especially in the North and Northeast regions, perhaps the result of more unfavorable changes in risk factors for diabetes in these regions. For example, over this time period, the risk-weighted prevalence (summary exposure value) of high BMI has risen at a greater rate in these regions than in the rest of the country.

This is the first report of national mortality trends by type of diabetes. The large decrease observed for age-standardized mortality for type 1 diabetes is consonant with a previous report of rapidly decreasing mortality due to acute complications of diabetes [19], which weighs more heavily in type 1 diabetes.

These findings can be contextualized in a world which was unprepared for the joint obesity and diabetes pandemics and has yet to discover how to effectively deal with them. What we report for Brazil can be found, in one degree or another, in most countries around the world. Diabetes, merely a curiosity in terms of international public health 40 years ago, being highly prevalent only in select native American and Pacific islander populations, is now a major and ever-growing concern [1]. The ongoing demographic, nutritional, and epidemiologic transitions will tend to exacerbate these trends in the future. Surprisingly, these GBD data show that the age-standardized incidence of type 2 diabetes in Brazil has remained basically stable over the period studied, despite a large increase in age-standardized high body mass index. The increases seen in diabetes prevalence over this period are thus mainly due to aging of the population and to the greater survival of those with the disease.

The geographic trends in estimated prevalence and burden in Brazil document that diabetes has increasingly shifted to the poor, as the Northeast and North regions are Brazil’s poorest. Data from a large Brazilian cohort study confirm this, showing a major educational gradient in prevalence, with those lacking a complete primary education having a 64% greater adjusted prevalence of diabetes than those with a university degree [18].

These findings demonstrate the urgent need for Brazilian society and health care organizations, especially the SUS (Sistema Único de Saúde, the Brazilian national health system), to develop effective strategies to stem the continued rise in diabetes prevalence and burden. These include both actions to prevent diabetes and actions to treat diabetes once present. The Brazilian Ministry of Health, responsible for maintaining the health of the population, must lead the way, aligning and prioritizing the use of available resources to control these major and growing vectors of disease burden. As population aging will continue, the incidence of diabetes must be decreased if the epidemic and its burden are to be controlled.

The 2011 Brazilian plan to confront the NCDs [20], consonant with the WHO Action Plan [21], emphasized the control of diabetes and many of its risk factors, but has yet to produce favorable trends in the prevalence, incidence, mortality, and burden of diabetes. On the positive side, recent findings from Vigitel suggest that the prevalence of obesity may have stabilized over the past 3 years [22], which, if confirmed, may eventually contribute to decrease burden.

As the focus of that plan, controlling risk factors, such as poor nutritional practices and sedentary lifestyles, appears appropriate and aligned with the World Health Organization [21] and what seems to be lacking is more effective implementation. Particularly, encouraging in terms of interventions which were implemented has been the 2014 Nutritional Guidelines for the Brazilian population [23, 24] as many recent studies have suggested an important role for ultraprocessed foods, focus of these Guidelines, in the current obesity pandemic [25, 26].

Clinical strategies stimulating lifestyle change in those at high risk to develop the disease have been shown to prevent diabetes [27, 28]. However, recent studies have made it clear that strategies focusing on the detection and treatment of those at high risk will be, by themselves, inadequate [29, 30]. Many reasons exist for this. One major one is that the diabetes “prevented” is in fact frequently merely “delayed” [31]. Added to this problem is the fact that, within the current social context, individual efforts to change lifestyle are frequently difficult and frustrating. Perhaps most importantly, type 2 diabetes is a life course disease—one that develops slowly during life [32, 33]. To focus only on high risk groups is to ignore the multitude of approaches to prevention that can be approached through population-based or primary care strategies—a healthy gestational lifestyle, breastfeeding, healthy nutrition, avoidance of weight gain in childhood, adolescence and early adulthood, and greater physical activity to name several major ones—which can be implemented prior to risk becoming acute. Finally, that over 50% of middle-aged and elderly Brazilians have been shown to be at high risk by at least one of the standard definitions of intermediate hyperglycemia [34], which adds an additional justification for promoting lifestyle changes in the whole population.

Most of these actions focus on preserving health rather than preventing disease and are population—rather than clinical-based in nature. They involve stimulating healthy choices, especially related to nutrition and physical activity [3, 21, 35]. Studies document the much greater effectiveness of population-based rather than clinical interventions for control of non-communicable diseases [36]. In terms of nutritional interventions, dozens have been recently proposed, and many were implemented in Brazil [37–39]; although with the changes over the past 2 years in the federal administration and the recession, much of what was initiated has come undone. Additionally, Brazilian society, like many around the world, has been in large part immobilized in forging population-level responses to this challenge. This is in part due to the relative novelty of public health approaches for chronic disease control. While Brazilian society has generally accepted that vaccinations, control of infectious disease vectors and well-baby measures, and even nutritional interventions of massive reach such as iodination of salt and water fluoridation, are within the accepted scope of government interventions, and it has yet to come to a similar consensus with respect to interventions aimed to minimize exposure to unhealthy foods and lifestyles. Reaching this consensus is complicated by negative inputs from economic interests, particularly the international food industry, which has adopted strategies of silently lobbying against such interventions while publicly casting doubt on the evidence base used for deeming products to be unhealthy, strategies similar to those used by tobacco companies in the recent past [40]. Additionally, if environmental pollutants do in fact have a role for in causing diabetes [41, 42], they must also receive due attention. In sum, recognition of the importance of such population-based interventions and work to implement them is urgently needed in Brazil.

Perspectives for the treatment of diabetes and its complications extrapolate the focus of this report. However, clinical trials have demonstrated that some of the more recently introduced anti-hyperglycemic medications have major benefit in terms of reduction of morbidity and mortality [43]. How best to incorporate these benefits into health care, especially the national health system, without bankrupting health care in the process, is a major challenge at present. Strict attention to providing other, more accessible treatments, such as those for hypercholesterolemia and hypertension, and guaranteeing that treatment goals are achieved, is another challenge, one especially relevant to primary care.

Limitations to this report merit discussion. Brazilian mortality data have weaknesses, including high degrees of incompleteness and of poor definition of cause, increasing risk of inaccuracy of historical trends. Access to medical care and quality of public health indicators are worse in the North and Northeast regions, making estimates for states of these regions particularly limited. Happily, these regional inequalities have diminished greatly over the past two decades. However, while incompleteness has diminished substantially, the definition of cause of death still presents an enormous degree of inaccuracy. As mentioned above, data on prevalence are based many on studies of small communities, which, even when pooled, are questionably representative of the Brazilian population. Further, data on the incidence of diabetes are particularly scarce, making estimates of incidence and its trends especially difficult. Another potential problem is that estimates of burden due to high fasting plasma glucose are based on relative risks developed from international data which may not represent the Brazilian context. Though the GBD has adopted approaches to minimize these problems, they have not been eliminated. Another limitation is that the forecasting estimates through 2040 have very broad uncertainty intervals. Finally, estimates of the diabetes and hyperglycemia disease burdens are complicated by difficulties in apportioning cause in diseases with multiple causes. Within this context, about 40% of deaths due to diabetes are currently believed to result from non-vascular complications, most of which have not been taken into consideration in GBD 2017 calculations. These complications include many types of cancer, chronic obstructive pulmonary disease, and pneumonia and other infections [44], and their omission may result in substantial underestimation of the diabetes burden. That said, the sophisticated, standardized approaches implemented by the Institute of Health Metrics and Evaluation, actively supported by Brazilian demographers, epidemiologists and statisticians, make these GBD 2017 estimates the best ones available to date and should permit that the doubts raised here will be resolved in future iterations of the GBD.

## Conclusions

The large and growing disease burden due to diabetes and high fasting plasma glucose in Brazil is projected to expand even further in future decades. Actions by the Brazilian national health system and the Brazilian society in general to curb this rise have been insufficient considering the size of current and projected disease burden. A major effort on preventing diabetes is needed.

## Supplementary information

**Additional file 1: Figure S1.** Dimensions of assessment of the disease burden of diabetes and high fasting plasma glucose, and burden attributable to its risk factors in the Global Burden of Disease 2017 (GBD 2017) study.

## Data Availability

Data we used in this article are publicly available online on the official website of Institute of Health Metrics and Evaluation (http://ghdx.healthdata.org/gbd-results-tool).

## Abbreviations

BMI: Body mass index
DALYs: Disability-adjusted life years
GBD: Global Burden of Disease
ICD: International classification of diseases
OGTT: Oral glucose tolerance test
PNAD: Pesquisa Nacional por Amostra de Domicílios
SUS: Sistema Única de Saúde
YLDs: Years lived with disability
YLLs: Years of life lost
WHO: World Health Organization
