# Supplementary material for: The burden of diabetes and hyperglycemia in Brazil: a global burden of disease study 2017
Source: Popul Health Metr. 2020 Sep 30;18(Suppl 1):9. doi: 10.1186/s12963-020-00209-0 (PMC7526086; doi:10.1186/s12963-020-00209-0)
Supplement: Supplementary file 1 — Additional file 1: Figure S1. Dimensions of assessment of the disease burden of diabetes and high fasting plasma glucose, and burden attributable to its risk factors in the Global Burden of Disease 2017 (GBD 2017) study. [file 12963_2020_209_MOESM1_ESM.docx]

**Supplementary Figures**

Supplementary Figure 1. Dimensions of assessment of the disease burden of diabetes and high fasting plasma glucose, and burden attributable to its risk factors in the Global Burden of Disease 2017 (GBD 2017) study.
